# Balancing HIV testing efficiency with HIV case-identification among children and adolescents (2–19 years) using an HIV risk screening approach in Tanzania

**DOI:** 10.1371/journal.pone.0251247

**Published:** 2021-05-06

**Authors:** Gretchen Antelman, Michelle M. Gill, Ola Jahanpour, Roland van de Ven, Catherine Kahabuka, Asheri Barankana, Sharon Lwezaura, Naftali Ngondi, Alison Koler, Peris Urasa, Rhoderick Machekano

**Affiliations:** 1 Elizabeth Glaser Pediatric AIDS Foundation, Dar es Salaam, Tanzania; 2 Elizabeth Glaser Pediatric AIDS Foundation, Washington, DC, United States of America; 3 Elizabeth Glaser Pediatric AIDS Foundation, Dar es Salaam, Tanzania; 4 Department of Epidemiology and Biostatistics, The Institute of Public Health, Kilimanjaro Christian Medical University College, Moshi, Tanzania; 5 CSK Research Solutions, Dar es Salaam, Tanzania; 6 Pact, Dar es Salaam, Tanzania; 7 National AIDS Control Program, Ministry of Health, Community Development, Gender, Elderly and Children, Dodoma, Tanzania; 8 Department of Social Welfare, Ministry of Health, Community Development, Gender, Elderly and Children, Dodoma, Tanzania; 9 Pact, Washington DC, United States of America; St. Ambrose University, UNITED STATES

## Abstract

To optimize HIV testing resources, programs are moving away from universal testing strategies toward a risk-based screening approach to testing children/adolescents, but there is little consensus around what defines an optimal risk screening tool. This study aimed to validate a 12-item risk screening tool among children and adolescents and provide suggested fewer-item tool options for screening both facility out-patient and community populations by age strata (<10 and ≥10 years). Children/adolescents (2–19 years) with unknown HIV status were recruited from a community-based vulnerable children program and health facilities in 5 regions of Tanzania in 2019. Lay workers administered the screening questions to caregivers/adolescents; nurses enrolled those eligible for the study and tested all participants for HIV. For each screening item, we estimated sensitivity, specificity, positive predictive value and negative predictive value and associated 95% confidence intervals (CI). We generated a score based on the count of items with a positive risk response and fit a receiver operating characteristic curve to determine a cut-off score. Sensitivity, specificity, positive predictive value (PPV; yield) and number needed to test to detect an HIV-positive child (NNT) were estimated for various tool options by age group. We enrolled 21,008 children and adolescents. The proportion of undiagnosed HIV-positive children was low (n = 76; 0.36%; CI:0.29,0.45%). A screening algorithm based on reporting at least one or more items on the 10 to 12-item tool had sensitivity 89.2% (CI:79.1,95.6), specificity 37.5% (CI:36.8,38.2), positive predictive value 0.5% (CI:0.4,0.6) and NNT = 211. An algorithm based on at least two or more items resulted in lower sensitivity (64.6%), improved specificity (69.1%), PPV (0.7%) and NNT = 145. A shorter tool derived from the 10 to 12-item screening tool with a score of “1” or more on the following items: relative died, ever hospitalized, cough, family member with HIV, and sexually active if 10–19 years performed optimally with 85.3% (CI:74.6,92.7) sensitivity, 44.2% (CI:43.5,44.9) specificity, 0.5% (CI:0.4,0.7) PPV and NNT = 193. We propose that different short-tool options (3–5 items) can achieve an optimal balance between reduced HIV testing costs (lower NNT) with acceptable sensitivity. In low prevalence settings, changes in yield may be negligible and NNT may remain high even for an effective tool.

## Introduction

The Joint United Nations Programme on HIV/AIDS (UNAIDS) estimates that, globally, 81% of people living with HIV (PLHIV) knew their status and 67% were on treatment by the end of 2019, but access to testing and treatment for children and young people continues to lag behind [1]. Global estimates show almost half (46%) of children living with HIV under age 15 have not accessed treatment, a gap largely due to missed opportunities in diagnosing vertical infection [2, 3]. Although adolescent age-disaggregated data are not routinely reported, the treatment coverage of children under age 15 years in Tanzania is estimated to be 65% [2], but only 42% of HIV-infected adolescents age 15–24 are on treatment [4].

For more than a decade, HIV testing policies have largely promoted universal provider-initiated testing and counseling (PITC) aiming to reach high, if not universal testing coverage [5, 6]. This approach has been successful in tuberculosis (TB) and antenatal clinics (ANC) where technical support, health information system monitoring, and often additional resources have been provided. Additionally, adopting a program standard of opt-out PITC in ANC and TB settings has contributed substantially to near-universal testing coverage in those settings. Yet in other settings, particularly general out-patient services, reaching high PITC coverage has been challenging [7] due to several barriers related to resource limitations including personnel, training, infrastructure and commodities [8]. Thus, clinicians would often practice an informal risk screening, testing only those patients suspected of having AIDS-defining illness or risk behaviors [9]. Additional challenges around consent/guardianship, provider attitudes toward the likelihood of HIV infection, complexities of implementing robust index testing initiatives at scale, and the fact that some children may not be reached by index or other facility-based testing due to health utilization barriers have further limited testing coverage [10–12], and widened the treatment gap between children/adolescents and adults.

As countries come closer to reaching the first two UNAIDS goals of 95% of all HIV infections diagnosed and 95% of those diagnosed on ART, strengthening and scaling index contact tracing and testing to identify remaining undiagnosed infections has become a more compelling strategy [13]. Consequently, the United States President’s Emergency Fund for AIDS Relief (PEPFAR) has called for more efficient HIV testing strategies through two primary pathways. First, improve HTS efficiency by elevating index testing to the predominant testing modality, aiming to account for 30–75% of all new diagnoses. And second, to promote the use of HIV risk screening algorithms to determine testing eligibility among general out-patient and community-based populations, while preserving established opt-out approaches for antenatal and TB clinics [14]. Current World Health Organization (WHO) testing guidelines also recommend targeted HIV testing using a symptom screening approach for general populations in low HIV burden settings, defined as national HIV prevalence below 5% [15]. While WHO recommendations do not differentiate testing strategies according to HIV prevalence among adults versus children, adult HIV prevalence in Tanzania (5.5%), as in most sub-Saharan African countries, is higher compared to children/youth under 20 years of age (0.7%). Furthermore, there are also significant regional variations in Tanzania with HIV prevalence ranging from <1% and 11% [4]. These wide disparities in HIV burden within sub-populations highlight the importance of policies that promote, or allow for, differentiated case-finding strategies that may be applied in both facility and community settings, whereby effectiveness is ultimately measured by the number of PLHIV diagnosed [16].

The combined effect of low uptake of index/family testing [17, 18], barriers to early infant testing [2], and high numbers of patients attending out-patient services means that the majority of HIV-positive children/adolescents are still being identified through facility-based PITC [19]. While the PITC modality remains critically important to identifying undiagnosed children/adolescents and reducing the pediatric treatment gap, challenges remain in determining what testing strategies will yield the largest numbers of newly identified HIV infected children/adolescents with available resources. Expanding targeted testing to those who may be reached more efficiently in their communities as opposed to facilities may also be an under-utilized strategy to reduce the pediatric treatment gap.

With greater focus on the investment returns from HIV testing services (HTS), programs now monitor and evaluate various HTS modalities using metrics such as yield (proportion HIV-positive among those tested) and number needed to test (NNT) to diagnose one individual [20]. Use of these metrics has led to a growing interest in HIV risk screening algorithms that are both efficient and effective in early identification of undiagnosed HIV infection among children/adolescents. But there is little consensus around what defines an optimal HIV risk screening tool [21] and given the success of opt-out PITC for special populations (pregnant women, TB-infected), some advocate for the universal approach to HIV testing among children and adolescents who already face substantial barriers to accessing HTS [22]. Furthermore, the risk of missing or delayed diagnosis is unacceptable due to higher risk of rapid disease progression among children and onward transmission among sexually active adolescents and young adults [2, 20].

Aiming to optimize access and yield [23, 24], recent studies [25, 26] have built upon earlier research on HIV risk screening algorithms conducted largely before opt-out PITC testing was widely adopted [6, 11, 12, 27]. Bandason et al. [25] evaluated a 4-item tool among out-patient children/adolescents (6–15 years) reporting 80% sensitivity and 66% specificity. Moucheraud [26], targeting all children under 15 years admitted to in-patient wards in Malawi, added two new items to the Bandason 4-item tool and found a sensitivity of 84% and specificity of 40%. Our study aimed to expand the evidence of the Bandason tool’s validity in a low prevalence setting and evaluate a 12-item HIV risk screening tool developed for community case workers to determine which beneficiaries of a community-based vulnerable children/adolescent program to refer for HIV testing. During data collection, the study protocol was amended to include a facility-based population with a goal of validating the 12-items of the tool developed for the community-based screening of children/adolescents and providing suggested fewer-item tool options for use in community or facility-based targeted (“optimized”) PITC. Findings from this study are expected to provide further evidence that HIV risk screening among children and adolescents can improve HIV testing resource use by applying an algorithm that limits access to HIV testing to those who report specific symptoms or risk factors.

## Methods

Data were collected January-September 2019 in two populations: Community-based families (households) enrolled in an orphan and vulnerable children’s (OVC) program and patients attending health facility out-patient departments (OPD) at participating health facilities. Children aged 2–9 years and adolescents aged 10–19 years comprised the target population. The study purposely selected communities to maximize the likelihood of recruiting undiagnosed positive children/adolescents and specifically aimed to recruit from households that had no program documentation of prior HIV risk screening/referral for testing of some or all children in that household. On average, we recruited two child/adolescent participants per household visited. A total of 19 geographic units (12 urban and 7 rural wards) were selected from four regions (Dar es Salaam, Njombe, Tabora, Shinyanga) for community-based study participation.

Our sample size aim was to recruit sufficient participants to yield 81 undiagnosed HIV-positive, but given the unexpectedly low prevalence of undiagnosed HIV, it proved impractical to achieve this within the study’s funding envelope. Thus, we added the facility population to the study to meet our sample size for tool validation metrics. Five health facilities in two regions (Tabora, Dodoma) were purposely selected because they had high patient volume and could feasibly nominate staff to be trained to perform screening, recruitment and data collection tasks. At facilities, the majority of participants were recruited from the main out-patient department (age 5–19 years) and the reproductive child health clinic (age 2–4 years).

The 12-item risk screening tool included four items validated by Bandason et al. [25], plus eight additional risk items hypothesized by the vulnerable children community program team to be related to HIV risk (see Box 1). The process by which the program finalized these screening items was iterative, through discussion with Government counterparts overseeing the OVC program, implementing partner technical personnel, and feedback from earlier implementation rounds administering the tool (prior to this research study). In the final tool evaluated in this study, ten questions were asked to caregivers of children under 10 years, and an additional two questions about sexual activity and pregnancy were asked to adolescents (or their caregivers) 10 years and older. The lay cadre administering the screening items were trained to request privacy from non-participants for the whole set of questions. Otherwise, there were no strict guidelines regarding who should be present and to whom questions (or certain questions) should be asked. The lay cadre typically negotiated this with each household depending on their unique situation and beneficiary/caregiver preference, and especially for the questions related to sexual activity. Consenting adolescents (emancipated minors and those over 18 years), however, were screened without a caregiver present (unless requested by the adolescent).

**Box 1. HIV risk screening items on 12-item tool.**

$$\begin{array}{ll} \begin{array}{l} \underline{\text{Bandason}\;\text{items}}\\ 1.\;\text{Are}\;\text{one}\;\text{or}\;\text{more}\;\text{siblings}\;\text{or}\\ \text{biological}\;\text{parents}\;\text{of}\;\text{the}\\ \text{child/adolescent}\;\text{deceased}\\ (relative\;died)?\\ 2.\;\text{Has}\;\text{the}\;\text{child/adolescent}\;\text{ever}\\ \text{been}\;\text{admitted}\;\text{to}\;\text{hospital}\\ \text{before?}\;(hospitalized)\\ 3.\;\text{Has}\;\text{the}\;\text{child/adolescent}\;\text{had}\\ \text{poor}\;\text{health}\;\text{in}\;\text{the}\;\text{last}\;3\\ \text{months}\;(poor\;health)?\\ 4.\;\text{Does}\;\text{the}\;\text{child/adolescent}\;\text{have}\\ \text{recurring}\;\text{skin}\;\text{problems}\;(skin)? \end{array} & \begin{array}{l} \underline{\text{Risk}\;\text{screening}\;\text{items}}\\ 5.\;\text{Is}\;\text{the}\;\text{child/adolescent}\;\text{living}\;\text{with}\;\text{a}\;\text{chronically}\;\text{ill}\;\text{parent}\;\text{or}\;\text{family}\;\\ \text{member}\;(family\;member\;ill)?\\ 6.\;\text{Has}\;\text{the}\;\text{child/adolescent,}\;\text{or}\;\text{anyone}\;\text{in}\;\text{the}\;\text{household}\;\text{ever}\;\text{been}\\ \text{prescribed}\;\text{TB}\;\text{treatment}\;(history\;TB)?\\ 7.\;\text{Are}\;\text{one}\;\text{or}\;\text{more}\;\text{siblings}\;\text{or}\;\text{biological}\;\text{parents}\;\text{of}\;\text{the}\;\\ \text{child/adolescent}\;\text{HIV}\;\text{positive}\;(family\;member\;HIV)?\\ 8.\;\text{Is}\;\text{the}\;\text{child/adolescent}\;\text{malnourished}\;(malnourished)?\\ 9.\;\text{Has}\;\text{the}\;\text{child/adolescent}\;\text{had}\;\text{a}\;\text{cough}\;\text{for}\;\text{one}\;\text{month}\;\text{of}\;\text{more}\;\\ (cough)?\\ 10.\;\text{Has}\;\text{the}\;\text{child/adolescent}\;\text{ever}\;\text{been,}\;\text{or}\;\text{is}\;\text{currently}\;\text{being}\;\text{abused}\;\\ (abuse)?\\ 11.\;(If\geq 10\;years)\;\text{Is}\;\text{the}\;\text{adolescent}\;\text{sexually}\;\text{active}\;(history\;sex)?\\ 12.\;(If\geq 10\;years)\;\text{Does}\;\text{the}\;\text{adolescent}\;\text{have}\;\text{a}\;\text{child}\;\text{of}\;\text{his/her}\;\text{own}\;\text{or}\;\\ \text{is}\;\text{pregnant}\;(history\;pregnancy)? \end{array} \end{array}$$

In both populations, the HIV risk screening tool was administered by trained non-medical people. Lay case workers assigned to work in their communities were trained by the OVC program team. Lay counselors or community health workers based at study facilities received training in using the tool from the study team. The lay cadre completed a paper checklist for each child screened composed of a screening eligibility section followed by the 12-item HIV risk screening questionnaire. The screening eligibility questions included age/date of birth, history of biological mother’s HIV status, current child breastfeeding status, and most recent HIV result if ever tested. Completed risk screening tool data were entered into an electronic study database by research nurses for the community-based OVC participants and by data clerks at the study facilities.

In both community and facility settings, the lay cadre completed the HIV risk screening tool first, and then trained research nurses confirmed study eligibility and obtained written informed consent for study participation from caregivers of children/adolescents under 18 years, or from adolescents aged 15–17 years and emancipated or those 18–19 years. Verbal assent from non-emancipated minors 10–17 years was obtained after consent from the caregiver. Study exclusion criteria included a history of antiretroviral therapy (ART; excluding antiretroviral prophylaxis), age under 5 years and tested HIV-negative after breastfeeding cessation, or age 5 years or older and tested HIV-negative in the past 6 months with no reported HIV exposure (sex/blood/needles) since their last HIV test. All of those screened, regardless of whether they answered positively to any of the 12 screening items, were recruited into the study and received HIV testing if they provided informed consent and did not have one of the above exclusion criteria. In both populations, the research nurses administered a short questionnaire using an electronic tool and documented the HIV test result after testing and counseling. The questionnaire, lasting about 5 minutes, assessed some demographics, HIV testing history, maternal and other family member HIV status, and attitudes toward HIV testing. It was administered privately with the consenting caregiver or adolescent. Study participants did not receive any compensation.

Among community participants, HIV testing was conducted in the home following national guidelines. For those diagnosed HIV positive in the community sample, a referral for HIV care was made by the research nurses who then actively followed-up linkage for those participants. In study facilities, facility nurses were trained in research procedures (consent, questionnaire administration), and they provided HTS, including post-test counseling and linkage to HIV care, as per the standard of care and part of their routine work in the facility.

### Statistical methods

We summarized participants’ demographic characteristics and responses to screening items using frequencies and proportions, disaggregated by source of study population. For each screening item, we estimated sensitivity, specificity, positive predictive value and negative predictive value and associated 95% confidence intervals using the *diagt* procedure (Stata V14.1, StataCorp, College Station, TX). For each individual, we calculated a score based on the total number of positive responses to the 12 items. We defined a screening tool based on a positive response to least one of the 12 items (the “full” tool). We fit a receiver operating characteristic (ROC) curve to assess the overall diagnostic ability of the tool and determined the best cut-off score using the Youden Index. We also defined the “Bandason” tool, based on reporting at least one positive response to the 4 Bandason items.

We then sought to develop optimal screening tools with a reduced number of screening items, by age groups. Criteria to optimize tools were decided a priori. These were to have as few items as possible while maintaining sensitivity over 80% and minimizing NNT. The following steps were taken to optimize screening tool models overall and by age group.

1. Using a computer-intensive variable selection technique, *gvselect* in Stata [28], models of different sizes were fit from the set of 12 items, starting with the best predictive model based on one variable and then progressively increasing the complexity of the models to find the best set based on Akaike Information criteria (AIC) and Bayesian Information criteria (BIC). The AIC maximizes the sensitivity and the BIC maximizes the specificity. The *diagt* command Stata [28] was used to generate validation measures for Stata-generated models, and any model yielding sensitivity > = 80% was shortlisted for optimization.
2. Logistic regression was used in a stepwise manner to identify all screening items associated with HIV status. Items associated at the p < .15 level were shortlisted for optimization.
3. Using all shortlisted items from the above statistical approaches, optimization was done manually by first defining “core” items that were common to models resulting from gvselect and logistic regression approaches, and then adding back in the remaining shortlisted items one-at-a-time.
4. Three final optimal models were identified for all ages and the two age strata.

All tools were developed using combined community and facility population groups. Participants with missing screening item responses were removed from analyses for models containing those items. We also estimated the proportion of participants screening eligible for HIV testing, defined as those with at least one or more positive responses to tool items divided by the total number with non-missing values. The NNT to find one HIV positive child/adolescent was estimated by dividing the number scoring at the cut-off threshold or higher on the defined tool by the number HIV-positive among those scoring at/above the cut-off on the tool.

The protocol received ethical approval from the local National Research Ethics Committee of the National Institute for Medical Research in Tanzania, and the Population Council Institutional Review Board based in the USA.

## Results

**Fig 1:**
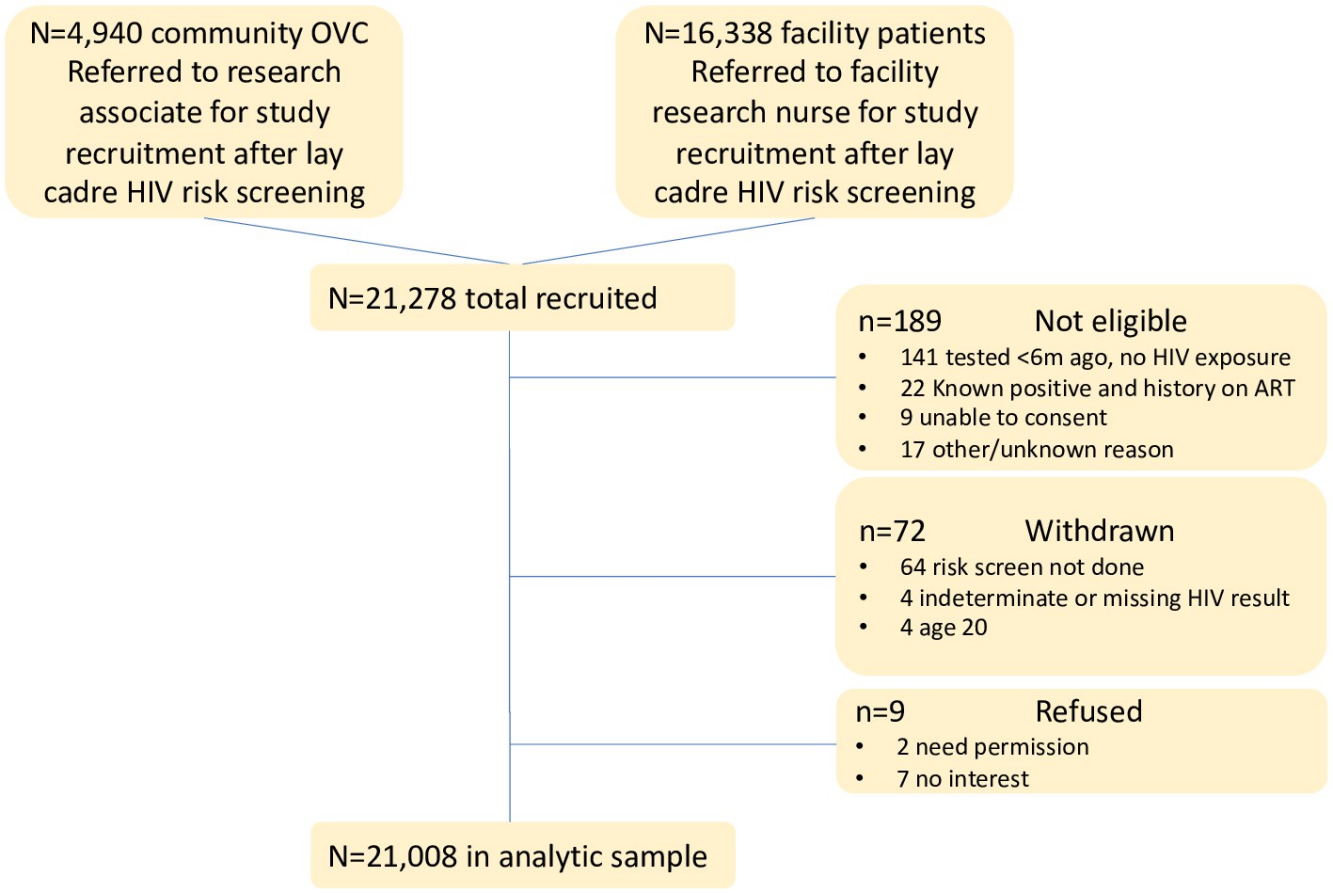
Study consort.

Among 21,278 children/adolescents recruited, a total of 21,008 (98.7%) were included in the analytic sample (n = 11,214 children age 2–9 years; n = 9794 adolescents age 10–19). We excluded 189 who were not eligible for the study, 72 who withdrew from the study, and 9 who did not provide consent (see Fig 1). Over three-quarters (77%, n = 16,151) were recruited from the facility and 23% (n = 4857) from the vulnerable children program in the community (Table 1). The proportion testing HIV positive was 0.36% (CI:0.29,0.45) and did not differ between community (0.43%; CI:0.27,0.66) and facility (0.33%; CI:0.25,0.44) groups (p = 0.22). About half (53%) of the participants were children under 10 years and 56% were female. Two-thirds (67%) were enrolled with their caregiver present; 22% had a history of HIV testing once or more, 10% reported that their mother had HIV (living or died).

**Table 1:** Demographic and risk characteristics of study participants by HIV status and sample source.

	Total		HIV positive			OVC-community^a^		OPD-facility^a^	
	N	%	N	%	95% CI	N	%	N	%
**Total enrolled**	21,008	100	76	0.36	(0.29,0.45)	4857		16,151	
OVC-community^a^	4857	23.1	22	0.45	(0.28,0.68)	—		**—**	
OPD-facility^a^	16,151	76.9	54	0.33	(0.25,0.44)	—		**—**	
**Age group**									
2 to 9	11,214	53.4	36	0.32	(0.22,0.44)	2330	48.0	8884	55.0
10 to 19	9794	46.6	40	0.41	(0.29,0.56)	2527	52.0	7267	45.0
**Sex**									
Male	9301	44.3	30	0.32	(0.22,0.46)	2283	47.0	7018	43.5
Female	11,707	55.7	46	0.39	(0.29,0.52)	2574	53.0	9133	56.5
**Emancipation status**									
Minor with caregiver	14,167	67.4	40	0.28	(0.20,0.38)	2860	58.9	11,307	70.0
Emancipated	4458	21.2	17	0.38	(0.22,0.61)	351	7.2	4107	25.4
Other	2383	11.3	19	0.80	(0.48,1.24)	1646	33.9	737	4.6
**History of HIV testing**									
Never	16,407	78.1	59	0.36	(0.27,0.46)	2693	55.4	13,714	84.9
Once	2885	13.7	13	0.45	(0.24,0.77)	1442	29.7	1443	1.0
Twice or more	1716	8.2	4	0.23	(0.06,0.60)	722	14.9	994	6.2
**HIV risk screening items**									
Ever hospitalized									
No	15,342	73.2	41	0.27	(0.19,0.36)	4219	87.1	11,123	69.1
Yes	5612	26.8	35	0.62	(0.43,0.87)	627	12.9	4985	30.9
Recurring skin condition									
No	19,916	94.9	60	0.30	(0.23,0.39)	4570	94.3	15,346	95.1
Yes	1067	5.1	16	1.48	(0.86,2.42)	278	5.7	789	4.9
Poor health last 3 months									
No	19,852	94.5	62	0.31	(0.24,0.40)	4514	93.0	15,338	95.0
Yes	1145	5.5	14	1.21	(0.67,2.04)	339	7.0	806	5.0
Malnourished									
No	20,721	98.8	67	0.32	(0.25,0.41)	4750	98.2	15,971	98.9
Yes	257	1.2	8	3.02	(1.35,6.04)	85	1.8	172	1.1
Cough ≥1 month									
No	20,254	96.5	65	0.32	(0.25,0.41)	4446	91.7	15,808	97.9
Yes	734	3.5	10	1.34	(0.65,2.49)	403	8.3	331	2.1
Abused (history/current)									
No	20,731	98.9	75	0.36	(0.28,0.45)	4735	98.1	15,996	99.2
Yes	228	1.1	1	0.44	(0.01,2.42)	93	1.9	135	0.8
TB in household									
No	19,579	93.4	63	0.32	(0.25,0.41)	4137	85.7	15,442	95.7
Yes	1384	6.6	13	0.93	(0.50,1.61)	690	14.3	694	4.3
Chronic ill family member									
No	19,538	93.1	65	0.33	(0.26,0.42)	4162	86.0	15,376	95.2
Yes	1446	6.9	11	0.75	(0.38,1.36)	679	14.0	767	4.8
Biological relative HIV									
No	18,739	89.8	54	0.29	(0.22,0.38)	3828	80.4	14,911	92.6
Yes	2130	10.2	20	0.93	(0.57,1.45)	935	19.6	1195	7.4
Biological relative died									
No	17,371	82.9	51	0.29	(0.22,0.29)	3441	71.3	13,930	86.4
Yes	3590	17.1	24	0.66	(0.43,0.99)	1388	28.7	2202	13.6
**Sexual activity (10–19 only)**									
Not sexually active	4800	52.9	18	0.37	(0.22,0.59)	1684	88.0	3116	43.5
Sexually active	4277	47.1	18	0.42	(0.25,0.66)	230	12.0	4047	56.4
Missing	717		4	0.55	(0.15,1.42)	613		104	
**Has a child/pregnancy (10–19 only)** ^b^									
No history child	7414	85.2	26	0.35	(0.22 0.51)	1474	95.8	5940	82.9
Has child/pregnant	1286	14.8	9	0.69	(0.32,1.32)	64	4.2	1222	17.1
Missing	1094		5	0.45	(0.15,1.06)	989		105	

^a^ OVC = orphan or vulnerable child; OPD = out-patient department.

^b^ Question applicable to males and females (“Does the adolescent have a child of his/her own, or is pregnant?”).

The majority of the risk screen items were associated with higher risk of HIV infection, with the exception of abuse, family member illness, sexual activity and history of pregnancy (Table 1). Items associated with the highest HIV-positivity were recurring skin conditions, poor health in the past 3 months, malnourishment, and cough for ≥1 month (Table 1). Table 2 shows the validation measures for the individual items. Items with the highest proportion reporting”yes” (>15%) in the population showed higher sensitivity (Sn) scores (hospitalized Sn = 46.1%; sexual activity Sn = 50.0%; relative died Sn = 32.0%) and lower specificity (Sp; 73.3%, 52.9% and 82.9% respectively) compared to the lower prevalence items.

**Table 2:** Sensitivity, specificity, PPV and NPV of individual risk screening items, all among children and adolescents 2–19 years (N = 21,008), and by age group (<10, ≥10).

	Sensitivity		Specificity		Positive Predictive Value		Negative Predictive Value	
	%	95% CI	%	95% CI	%	95% CI	%	95% CI
**Risk screening items, 2–19 years**								
Ever hospitalized	46.1	(34.5,57.9)	73.3	(72.7,73.9)	0.6	(0.4,0.9)	99.7	(99.6,99.8)
Poor health last 3 months	18.5	(10.5,29.0)	94.6	(94.3,94.9)	1.2	(0.7,2.0)	99.7	(99.6,99.8)
Recurring skin condition	21.1	(12.5,31.9)	95.0	(94.7,95.3)	1.5	(0.9,2.4)	99.7	(99.6,99.8)
Malnourished	10.7	(4.7,19.9)	98.8	(98.7,99.0)	3.1	(1.4,6.0)	99.7	(99.6,99.8)
Cough ≥1 month	13.3	(17.4,38.6)	91.9	(82.4,83.4)	9.7	(0.4,1.0)	99.7	(99.6,99.8)
Abused (history/current)	1.3	(0.0,7.1)	98.9	(98.8,99.0)	0.4	(0.0,2.4)	99.6	(99.5,99.7)
TB in household	17.1	(9.4,27.5)	93.4	(93.1,93.8)	0.9	(0.5,1.6)	99.7	(99.6,99.8)
Chronic ill family member	14.5	(7.5,24.4)	93.1	(92.8,93.5)	0.8	(0.4,1.4)	99.7	(99.6,99.8)
Biological relative HIV	27.0	(17.4,38.6)	89.9	(89.4,90.3)	0.9	(0.6,1.4)	99.7	(99.6,99.8)
Biological relative died	32.0	(21.7,43.8)	82.9	(82.4,83.4)	0.7	(0.4,1.0)	99.7	(99.6,99.8)
Sexually active, age ≥10	50.0	(32.9,67.1)	52.9	(51.9,52.9)	0.4	(0.2,0.7)	99.6	(99.4,99.8)
Child/pregnant, age ≥10	25.7	(12.5,43.4)	85.3	(84.5,96.0)	0.7	(0.3,1.3)	99.6	(99.5,99.8)
**2–9 years**								
Ever hospitalized	50.0	(32.9,67.1)	72.2	(71.3,73.0)	0.6	(0.3,0.9)	99.8	(99.6,99.9)
Poor health last 3 months	19.4	(8.2,36.0)	93.9	(93.4,94.3)	1.0	(0.4,2.1)	99.7	(99.6,99.8)
Recurring skin condition	22.2	(10.1,39.2)	93.3	(92.8,93.8)	1.1	(0.5,2.1)	99.7	(99.6,99.8)
Malnourished	22.9	(10.4,40.1)	98.5	(98.3,98.7)	4.5	(2.0,8.8)	99.8	(99.6,99.8)
Cough >1 month	14.3	(4.8,30.3)	96.3	(95.9,96.6)	1.2	(0.4,2.7)	99.7	(99.6,99.8)
Abused (history/current)	2.9	(0.1,14.5)	99.4	(99.2,99.5)	1.4	(0.0,7.7)	99.7	(99.6,99.8)
TB in household	19.4	(8.2,36.0)	93.7	(93.2,94.1)	1.9	(0.4,2.0)	99.7	(99.6,99.8)
Chronic ill family member	22.2	(10.1,39.2)	93.8	(93.3,94.2)	1.1	(0.5,2.2)	99.7	(99.6,99.8)
Biological relative HIV	32.4	(17.4,50.5)	89.3	(88.7,89.9)	0.9	(0.5,1.6)	99.8	(99.7,99.9)
Biological relative died	34.3	(19.1,52.2)	86.0	(85.4,86.7)	0.9	(0.4,1.3)	99.8	(99.6,99.8)
**10–19 years**								
Ever hospitalized	42.5	(27.0,59.1)	74.6	(73.7,75.4)	0.7	(0.4,1.1)	99.7	(99.5,99.8)
Poor health last 3 months	17.5	(7.3,32.8)	55.5	(95.0,95.9)	1.6	(0.6,3.2)	99.6	(99.5,99.8)
Recurring skin condition	20.0	(9.1,35.6)	96.9	(96.5,97.2)	2.6	(1.1,5.0)	99.7	(99.5,99.8)
Malnourished	0.0	(0.0,8.8)	99.2	(99.0,99.3)	0.0	(0.0,4.5)	99.6	(99.4,99.7)
Cough >1 month	12.5	(4.2,26.8)	96.8	(96.5,97.2)	1.6	(0.5,3.7)	99.6	(99.5,99.7)
Abused (history/current)	0.0	(0.0,8.8)	98.4	(98.1,98.6)	0.0	(0.0,2.3)	99.6	(99.4,99.7)
TB in household	15.0	(5.7,29.8)	93.1	(92.6,93.6)	0.9	(0.3,1.9)	99.6	(99.5,99.7)
Chronic ill family member	7.5	(1.6,24.0)	92.4	(91.8,92.9)	0.4	(0.1,1.2)	99.6	(99.4,99.7)
Biological relative HIV	22.5	(10.8,38.5)	90.5	(89.9,91.1)	1.0	(0.4,1.8)	99.6	(99.5,99.8)
Biological relative died	30.0	(16.6,46.5)	79.4	(78.6,80.2)	0.7	(0.3,1.3)	99.6	(99.5,99.8)
Sexually active, age >10	50.0	(32.9,67.1)	52.9	(51.9,53.9)	0.4	(0.2,0.7)	99.6	(99.4,99.8)
Child/pregnant, age ≥10	25.7	(12.5,43.4)	85.2	(84.5,86.0)	0.7	(0.3,1.3)	99.6	(99.5,99.8)

**Fig 2:**
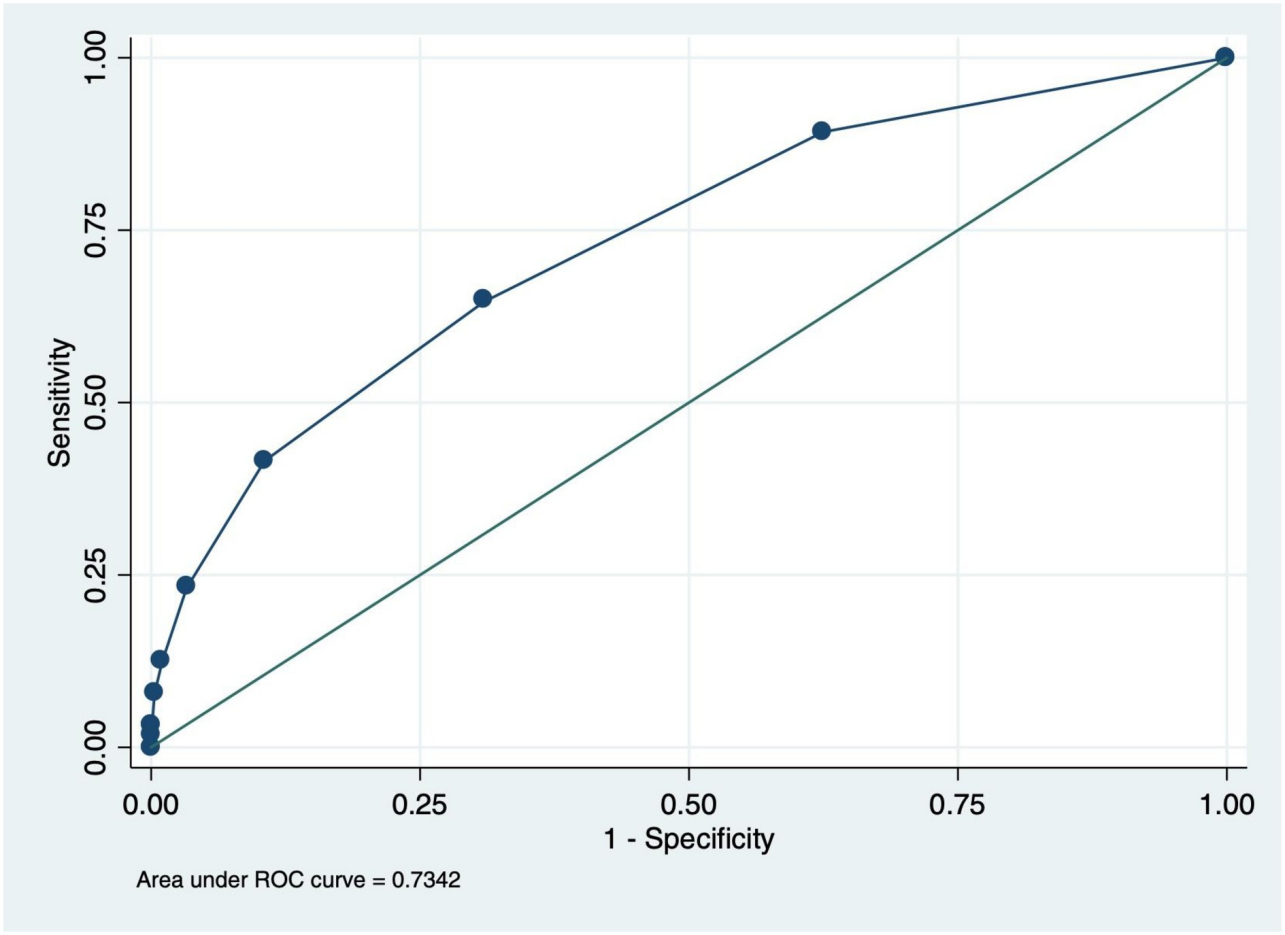
Receiver operating characteristic curve for each cut-off for the full (12) item screening tool, ages 2–19 years.

Table 3 shows the diagnostic performance of various proposed screening tools. The area under the ROC curve based on the 12 items was 0.734 (Fig 2). The Youden index estimates suggested that a cut-off score of 2 among all items would yield a more balanced tool (64.6% sensitivity, 69.1% specificity) with a NNT equal to 145. A tool based on scoring positive on at least one item from all the 12 items (full-item tool) had estimated sensitivity of 89.2% (CI: 79.1, 95.6), specificity of 37.5% (CI: 36.8, 38.2) and PPV of 0.5% (CI: 0.4, 0.6) with almost two-thirds (63%) of the children and adolescents screening eligible for testing. The estimated NNT was 211. The 4-item Bandason tool, with a cutoff of “1”, had similar sensitivity (64.5%) as the full tool with a cut-off of “2”, lower specificity (57.5%) and higher NNT (183).

**Table 3:** Validation measures for different risk screening tool options, by age group.

Tool model and items	Age group (years)	N screened^a^	N (%) screen eligible for HIV test	Sensitivity		Specificity		PPV		NPV		NNT
				%	95% CI	%	95% CI	%	95% CI	%	95% CI	
**All items** ^b^												
Score of ≥1 among all tool items	All age (2–19)	19,570	12,243 (63%)	89.2	(79.1,95.6)	37.5	(36.8,38.2)	0.5	(0.4,0.6)	99.9	(99.8,100)	211
	<10 (2–9)	11,061	5870 (53%)	87.1	(70.2,96.4)	47.0	(46.1,48.0)	0.5	(0.3,0.7)	99.9	(99.8,100)	217
	≥10 (10–19)	8509	6373 (75%)	91.2	(76.3,98.1)	25.2	(24.2,26.1)	0.5	(0.3,0.7)	99.9	(99.6,100)	206
Score of ≥2 among all tool items	All age (2–19)	19,570	6078 (31%)	64.6	(51.8,76.1)	69.1	(68.4,69.7)	0.7	(0.5,0.9)	99.8	(99.7,99.9)	145
	<10 (2–9)	11,061	2309 (21%)	54.8	(36.0,72.7)	79.2	(78.5, 08.0)	0.7	(0.4,1.2)	99.8	(99.7,99.9)	136
	≥10 (10–19)	8509	3769 (44%)	73.5	(55.6,87.1)	55.8	(54.8,56.9)	0.7	(0.4,1.0)	99.8	(99.6,99.9)	151
**Bandason (score ≥1)**												
Relative died Hospitalized	All age (2–19)	20,884	8943 (43%)	64.5	(52.7,75.1)	57.5	(56.8,58.1)	0.5	(0.4,0.7)	99.8	(99.7,99.9)	183
Poor health	<10 (2–9)	11,170	4764 (43%)	71.4	(53.7,85.4)	57.5	(56.5,58.4)	0.5	(0.3,0.8)	99.8	(99.7,99.9)	191
Skin	≥10 (10–19)	9714	4139 (43%)	60.0	(43.3,75.1)	57.5	(56.5,58.5)	0.6	(0.4,0.9)	99.7	(99.5,99.8)	172
**Optimized (score ≥1)**												
Relative died	All age (2–19)	20,069	11,219 (56%)	85.3	(74.6,92.7)	44.2	(43.5,44.9)	0.5	(0.4,0.7)	99.9	(99.8,99.9)	193
Hospitalized												
Family HIV												
Cough												
History sex ^c^												
Relative died	<10 (2–9)	11,129	5018 (45%)	81.8	(64.5,93.0)	55.0	(54.1,55.9)	0.5	(0.4,0.8)	99.9	(99.8,100)	186
Hospitalized												
Family HIV												
Poor health												
Hospitalized	≥10 (10–19)	9028	5438 (60%)	88.9	(73.4,96.9)	39.9	(38.9,40.9)	0.6	(0.4,0.8)	99.8	(99.7,100)	170
History sex ^c^												
Cough												

^a^ Number respondents with non-missing response to all items in respective tool.

^b^ Children under 10 years responded to 10 items; adolescents ≥10 years responded to 12 items.

^c^ Adolescents over 10 years with missing responses to history or sex or child/pregnancy were excluded from the analysis of the full 12-item model; those under 10 years were coded as “0” for history pregnancy/sex.

If a single tool were to be used for all age groups, a 5-item lay-administered tool containing two of the Bandason items (relative died, ever hospitalized), and additional items (cough, family member with HIV, and sexually active if 10–19 years) performed optimally with 85.3% (CI: 74.6, 92.7) sensitivity, Sp = 44.2%, PPV = 0.5%, and NNT = 193.

In the age group-stratified analyses and using a cut-off score of “1”, a 4-item tool was identified for children age 2–9 and a 3-item tool for adolescents age 10–19 years. For those under 10 years, relative died, history of hospitalization, family member with HIV and “poor health in the past 3 months” had 81.8% sensitivity, 55.0 specificity, 0.5% PPV, and 186 NNT. For adolescents, a 3-item tool containing history of hospitalization, being sexually active and “cough for > = 1 month” had 88.9% sensitivity, 39.9% specificity, 0.6% PPV and170 NNT) with 60% of those screened being eligible for testing.

## Discussion

We identified three lay-administered short tool options for optimized HIV risk screening in community and facility settings. In program settings, preferring to use the same tool for children and adolescents (age <20 years), a 5-item tool had a sensitivity of 85% and nearly halved (56%) the number who would undergo HIV testing. Use of this short tool could decrease the number needed to test per HIV diagnosis from 264 to 193, but it would also miss 15% of truly HIV positive children/adolescents who could have been diagnosed and put onto treatment under universal testing conditions. For screening programs able to deploy tools tailored to age group, a 4-item tool for those under 10 years showed 82% sensitivity and 3-item tool for those 10–19 years showed 89% sensitivity.

The full 12-item risk screening tool, with a cut-off score of “1”, had comparable sensitivity (89%) to the shorter 5-item tool but was not very efficient due to the larger proportion of those screened who would be referred for testing. Raising the cut-off score to “2” provided optimal testing efficiency but would miss an unacceptably high proportion (36%) of HIV-positive children/adolescents. Implementation costs associated with a longer tool, though not measured in this study, would likely be more compared to a shorter tool due to training, monitoring, staff time and the likelihood that a larger proportion would screen eligible for HIV testing. In addition, a long or complex tool could lead to provider short-cuts and loss of fidelity/standardization. The previously validated lay-administered Bandason risk screening tool which achieved 80% sensitivity among children age 6–15 in Zimbabwe [25] did not perform as well in Tanzanian children 2–19 years with a sensitivity of only 65%, although performance was less divergent compared to those under age 10 (sensitivity: 71%). This suggests that risk factors among adolescents are likely to be different from children, and different items or branching algorithms for those above and below cut-off ages of 10 or 15 years may be indicated.

These findings are consistent with prior studies which have also found that relatively few risk screening items can be predictive of HIV positivity [12, 26, 29]. Furthermore, the tools identified in this study contain similar or identical items found to be useful in other studies, such as a relative having died or ever-hospitalized. However, despite these similarities, validation metrics may not be replicable across studies as they are influenced by variations in prevalence in risk factors across populations, overall HIV prevalence and the possibility that some risk factors could have varying levels of association with HIV. In Malawi, for example, “ever hospitalized” was found to be a less specific predictor of HIV status due to high rates of hospitalization for malaria [26]. And in our study, the NNT estimates were much higher than what other studies have reported [12, 26, 29] due to the extremely low prevalence of undiagnosed HIV among children/adolescents in Tanzania (fewer than 4/1000).

The composition of the recommended 5-item tool (for all ages/settings) can be broken into three sections: two items relate to a general history of illness or an elevated risk due to possible TB-HIV co-infection (hospitalized, cough): two items assess risk of vertical exposure (relative died, family member HIV); and one item assesses risk of horizontal exposure among adolescents (sexual activity). Efforts to increase coverage of index contact HTS have intensified recently to ensure all biological siblings, offspring and sexual partners of known HIV positive individuals are offered HIV testing [13, 14, 17]. These efforts usually take place within HIV clinic settings, are limited to contacts of known (and in-treatment) HIV-positive individuals [21], and can be complex interventions to administer due to provider, resource and patient-related barriers [13]. This study shows that an effective screening tool with only two questions assessing index contact exposure may augment HIV case-identification among community-based and out-patient children/adolescents who are not currently identified through index contact tracing. Implementing a validated screening tool within PITC initiatives, at facilities and through community programs, can optimize PITC as a complementary strategy for reaching undiagnosed children/adolescents. Indeed, Yumo et al. [9] concluded that the combined model of symptom-based screening plus index testing was more efficient in yielding comparable numbers of HIV-positive children/adolescents diagnosed as compared to universal PITC, most likely because truly universal testing is difficult to achieve. However, a major limitation of symptom-based screening questions is that they will miss asymptomatic cases, suggesting the need for continued exploration of potential additional items that capture HIV risk factors with higher sensitivity.

We must be cautious generalizing research-derived validation measures to program settings, where the actual implementation of a standardized or validated screening algorithm may be quite different from research conditions. In Tanzania, for example, implementing partners have supported the roll-out of the national draft risk screening tool to optimize PITC in out-patient settings. The tool is used as a job aid, whereby providers and lay cadre are trained to follow an algorithm assessing age, testing history, and up to 16 screening questions to determine testing eligibility. Since eligible patients are only documented according to question category (e.g., HIV exposure, general health, tuberculosis or sexually transmitted infection signs/symptoms), it is likely that fidelity to the screening process varies substantially across settings and providers. Fortunately, all of the items appearing in recommended models in this study are included in the Tanzania tool, and a pragmatic evaluation of this tool, under program conditions, is underway. As use of risk screening tools is scaled up programmatically, continued monitoring of real-world validation metrics will be essential; not necessarily at item level, but to ensure that the optimized PITC approach delivers on its promise to maximize the number of children/adolescents diagnosed, even if this means favoring sensitivity over specificity and accepting lower-than-expected yield.

This study has a few important limitations. First, we found significantly lower prevalence of undiagnosed HIV than program data suggested, affecting our power in stratified analyses of pertinent age groupings (e.g., ages 10–14; <15; 15–19 years) or recruitment source (community, facility). As other validation studies are currently underway, meta-analyses of combined datasets may be fruitful, especially to inform policy in countries with lower HIV prevalence where country-specific validation studies could be too expensive to conduct. Second, while the tool assessed “family member with HIV,” the risk tool did not directly assess vertical transmission risk (biological mother having HIV) for those age 5–19 years and only assessed exposed infant status in the screening eligibility questions for those age 2–4 years (thus, the item was not used in model building). Based on known risks of missing early infant diagnostic services [19] this would be an important item for the lay-cadre to have assessed directly for all children up to age 10 or 14 years. Third, the study was not designed to sample populations that were representative of all children/adolescents in Tanzania and it is possible that sub-groups of at-risk children/adolescents were under-represented in our sample. However, given the large number of participants overall, plus the fact that the majority of children in Tanzania have access to facility-based HTS, and OVC programs have emphasized HIV testing of all beneficiaries through referral to facilities, we believe that our findings strongly suggest that undiagnosed HIV infection among children/adolescents in Tanzania is indeed very small. Fourth, accurate assessment of sexual activity among adolescents is challenging both in research and in practice, and while the sexual activity item was found to be important in final algorithms, we had a high proportion of missing responses to this item, and likely a high proportion of under-reporting sexual activity as well. Thus, continued innovation in how to improve the accuracy and completeness of sexual history reporting could improve the effectiveness of the screening tool. And finally, we did not measure cost parameters associated with integrating HIV risk screening to PITC, such as training, supervision, human resources, monitoring, reporting and patient opportunity (wait times, transport). Several studies have called for economic evaluations or cost-effectiveness studies to better inform the policy response to calls for increased HTS efficiencies [30].

## Conclusions

We found that a single 5-item short-tool option for children and adolescents ages 2–19 years, or age group-tailored 3 to 4-item tools, can achieve acceptable levels of sensitivity and would likely introduce moderate efficiencies to HIV testing services offered in facilities or through community programs targeting vulnerable children/adolescents. We argue that risk screening tools should be short, easily administered by lay cadre, and maximize sensitivity in order to minimize the number of missed diagnoses. In low prevalence settings, changes in yield may be negligible and NNT may remain high even for an optimal tool. Given that opt-out (universal) facility-based PITC has faced challenges in reaching high coverage and vulnerable populations in community programs may under-utilize health facilities, incorporating standardized HIV risk screening into out-patient or community HTS could significantly increase case detection of undiagnosed HIV among children and adolescents in two ways. First, a validated tool should improve the targeting of limited resources to those patients most at risk for HIV-infection. Second, the programmatic investments made to implement such a tool—training, management buy-in, supervision, data monitoring–could help to reduce barriers to HTS related to provider attitudes/misperceptions of patient risk or need for testing. But ultimately, whether to adopt a risk screening tool or not cannot be a one-size-fits-all recommendation. Policy-makers will need to look beyond screening validation metrics (sensitivity, specificity, yield, NNT) in order to balance efficiency in use of HIV test kits with their collective obligation to identify all HIV-infected children/adolescents as early as possible [20], taking into account program monitoring or survey data pointing to population sub-groups or service points with higher rates of undiagnosed HIV infection and their HTS resource envelope.
